# Silencing expression of the *Rhipicephalus microplus* vitellogenin receptor gene blocks *Babesia bovis* transmission and interferes with oocyte maturation

**DOI:** 10.1186/s13071-018-3270-1

**Published:** 2019-01-05

**Authors:** Hala E. Hussein, Wendell C. Johnson, Naomi S. Taus, Carlos E. Suarez, Glen A. Scoles, Massaro W. Ueti

**Affiliations:** 1 Department of Veterinary Microbiology and Pathology, Washington State University, Pullman, WA 99164 USA; 2 Department of Entomology, Faculty of Science, Cairo University, Giza, 12613 Egypt; 3 Animal Disease Research Unit, USDA-ARS, Washington State University, 3003 ADBF, P.O. Box 646630, Pullman, WA 99164 USA

**Keywords:** *Rhipicephalus microplus*, Vitellogenin receptor, *Babesia bovis*, RNA interference

## Abstract

**Background:**

*Rhipicephalus microplus* is an efficient biological vector of *Babesia bovis*, a causative agent of bovine babesiosis. *Babesia bovis* is passed transovarially to the next generation of ticks, which then transmit the parasite to naïve animals. Due to the importance of the *R. microplus* ovary for tick reproduction and transmission of *B. bovis*, we investigated the hypothesis that silencing vitellogenin receptor gene expression in the ovary during tick feeding on *B. bovis*-infected cattle would affect parasite transmission to the next generation of ticks.

**Results:**

Silencing expression of the vitellogenin receptor in the ovary by RNA interference, resulted in reduced tick fertility. We observed reduced egg production (i.e. reduced weight of eggs), a lower rate of embryonic development, and a reduction in hatching. Analysis of individual larvae by PCR confirmed that RNAi mediated downregulation of the *R. microplus* vitellogenin receptor and also interfered with transovarial transmission of *B. bovis*. None of the larvae (0/58) from the RmVgR dsRNA-injected group were PCR-positive, whereas 12% (7/58) and 17% (10/58) of larvae from the non-injected and buffer-injected control groups, respectively, were infected with *B. bovis*.

**Conclusions:**

The combined effects of reduced fecundity and reduced infection in surviving larvae resulting from silencing indicate that vitellogenin receptor is essential for tick reproduction and may play a vital role in *B. bovis* transmission.

## Background

*Rhipicephalus microplus* is a blood-feeding ectoparasite of great importance to animal health, causing significant economic losses to the livestock industry, both directly and by transmitting pathogenic agents including *Babesia bovis* [1–3]. Larvae of *R. microplus* attach to cattle and feed to repletion before molting to the nymphal stage. Nymphs feed to repletion on the same animal and molt to adult stages. After completing a final blood meal, fully engorged females drop off to lay their eggs in the environment. Oogenesis is the development of oocytes in the ovaries, while vitellogenesis is the process whereby yolk proteins, known as vitellogenin (Vg) are synthesized in extraovarian tissues, transported by hemolymph to the ovaries and deposited inside tick oocytes [4]. In hard ticks, Vg is a high molecular mass-precursor that is synthesized in the fat bodies and midgut [5–10]. Vg is released into the hemolymph and is taken up by oocytes *via* a receptor mediated endocytosis [11]. Vg accumulates inside oocytes yolk granules as vitellin, which is a critical nutrient for tick embryo development [12]. A vitellogenin receptor (VgR) gene has been identified in a variety of tick species including *Dermacentor variabilis* [13], *Haemaphysalis longicornis* [14], *Amblyomma hebraeum* [15]. *R. microplus* and *R. appendiculatus* [16].

The sequence of the VgR of *R. microplus* (RmVgR) gene contains an open reading frame of 5400 base pairs encoding a large protein of 1800 amino acids. The amino acid sequence of RmVgR has high similarity with VgR from *Ixodes scapularis*, *D. variabilis* and *A. hebraeum*. Silencing with RmVgR double stranded RNA (dsRNA) reduced VgR expression resulting in reduced fertility and a decrease in the number of larvae produced [16]. In a previous report, silencing of VgR affected transovarial transmission of *B. gibsoni* by female *H. longicornis* fed on infected dogs, as indicated by the absence of parasite DNA in the egg mass laid by the group injected with dsRNA [14]. Similar to *B. gibsoni*, *B. bovis* is transmitted transovarially to the next tick generation. It was unknown if silencing of RmVgR gene would affect transovarial transmission of *B. bovis*. To fill this knowledge gap, we tested the hypothesis that RNAi-mediated silencing interfered with tick fitness and affected *B. bovis* transovarial transmission to the next generation of ticks. This study enhances our understanding of the essential role of RmVgR in tick reproductive physiology, and offers an additional candidate target for the development of strategies to control the tick and *B. bovis* transmission.

## Results

**Fig. 1:**
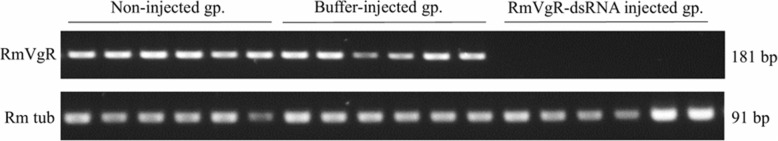
RmVgR dsRNA silences the transcription of vitellogenin receptor. Representative expression RmVgR gene using RT-PCR performed using cDNA from 6 ovary samples from each group (non-injected; buffer-injected; and RmVgR-dsRNA), dissected day one after female dropping. *Rhipicephalus microplus* α-tubulin was used as a positive control. Amplicons were separated in a 2% agarose gel

**Fig. 2:**
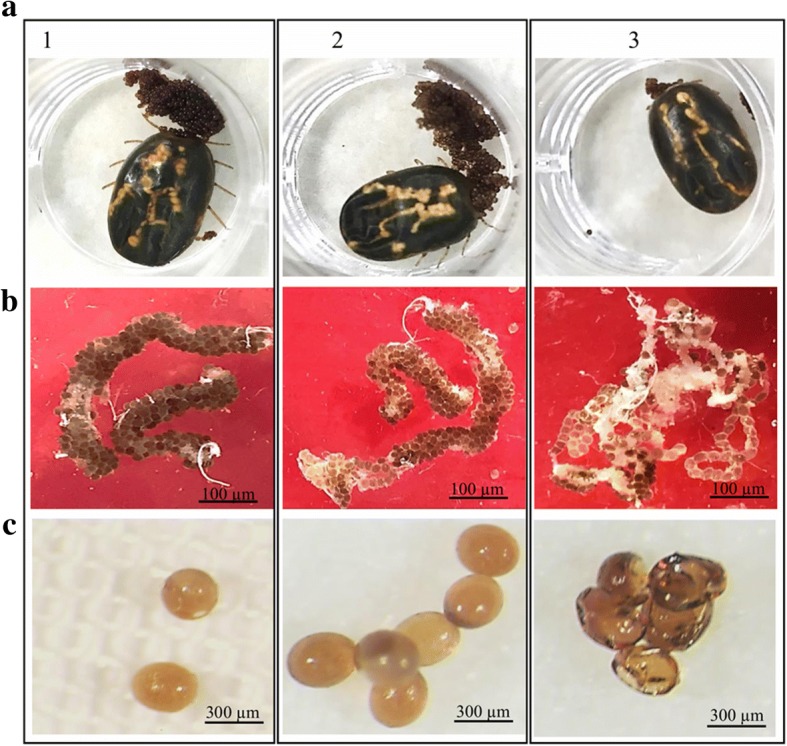
RmVgR dsRNA reduced the ability of female ticks to produce viable eggs. Fitness parameters of the treated groups are compared: 1, non-injected; 2, buffer control; 3, RmVgR dsRNA-injected. **a** Engorged female ticks. **b** Tick ovaries, dissected on day 7 after female dropping. **c** Tick eggs

Evaluation of RmVgR transcripts by RT-PCR demonstrated that transcripts of VgR were dramatically downregulated in tick ovaries derived from the RmVgR-dsRNA group. In contrast, RmVgR transcripts were detectable in both control groups (Fig. 1). *Rm α*-tubulin PCR was used as a positive control to demonstrate the presence of RNA transcripts in the tested samples. These results confirm that RmVgR-dsRNA effectively targeted and silenced transcription of the VgR. Tick phenotypes including tick engorgement, oviposition, and tick hatchability were also investigated in order to evaluate effects resulting from silencing expression of the vitellogenin receptor (Table 1). There was no significant difference in the adult survival rate of females after injection or in the weight of female ticks or in the number of female ticks fed to repletion between all three groups. The adult survival rate of females was 92% in non-injected group, 90% in buffer-injected group and 91% in RmVgR-dsRNA-injected group. The average weight of engorged 306.27 ± 5.21 mg for RmVgR-dsRNA-injected female ticks and 306.27 ± 6.42 mg for the buffer-injected group (*t*_(179)_ = 0. 6, *P* = 0.9996). The average weight of engorged non-injected female ticks was 306.25 ± 6.14 mg. The average weight of engorged non-injected female ticks was 306.25 ± 6.14 mg compared to RmVgR-dsRNA injected group (*t*_(181)_ = 1.7, *P* = 0.9986) (Table 1). Overall more than 90% of females from the three groups successfully fed to repletion (Table 1) (Fig. 2a). However, examination of ovary tissues confirmed that in the RmVgR-dsRNA group the ovaries were abnormal and there was a reduced number of ovarioles (Fig. 2b). Furthermore, females in the RmVgR-dsRNA group laid irregularly formed eggs (Fig. 2c).

**Table 1:** Effect of *Rhipicephalus microplus* VgR silencing on female ticks fed on a calf infected with *Babesia bovis*

Group	Engorged females	Engorged females weight (mean ± SE) (mg)	Oviposition rate	Egg weight (mean ± SE) (mg)	Egg diameter (mean ± SE) (mm)	Embryonic development rate (mean ± SE) (%)	Larvae survival rate (%)
Non-injected	92% (92/100)	306.25 ± 6.14	95% (57/60)	109 ± 4.32	0.379 ± 0.017	93.2 ± 6.40	100
Buffer	90% (90/100)	306.27 ± 6.42	98%(57/58)	121 ± 4.94	0.376 ± 0.012	92.2 ± 1.28	100
RmVgR-dsRNA	91% (91/100)	306.27 ± 5.21	91% (53/58)	42 ± 3.36*	0.256 ± 0.015*	39.1 ± 5.21*	100

Asterisks (*) indicate statistically significant differences with *P* < 0.0001 by comparing RmVgR group to buffer-control group or to non-injected group

**Fig. 3:**
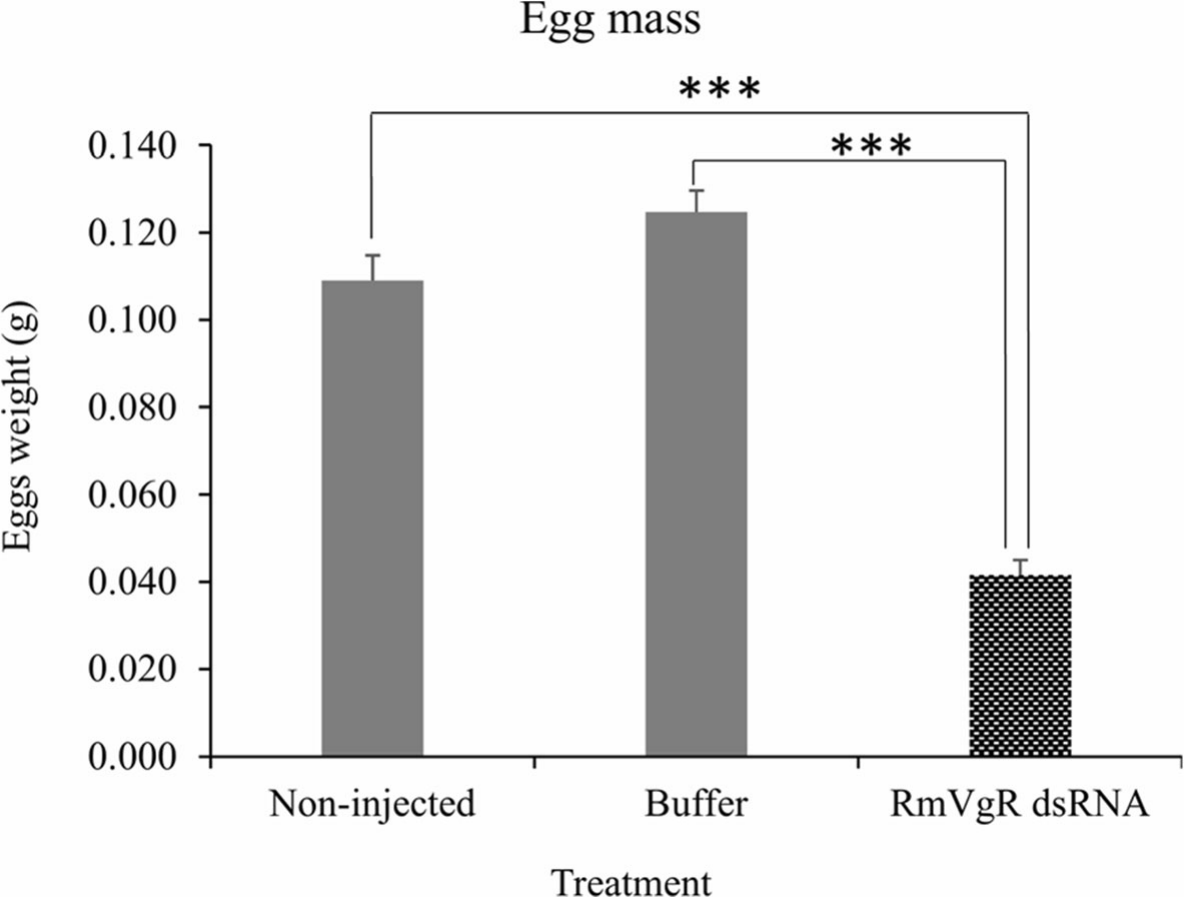
Silencing RmVgR reduced tick egg masses production. Asterisks indicate statistically significant differences: *t*_(114)_ = 13.4, *P* < 0.0001 (RmVgR-dsRNA-injected group *vs* buffer-injected group); and *t*_(111)_ = 10.22, *P* < 0.0001 (RmVgR-dsRNA-injected group *vs* non-injected group)

**Fig. 4:**
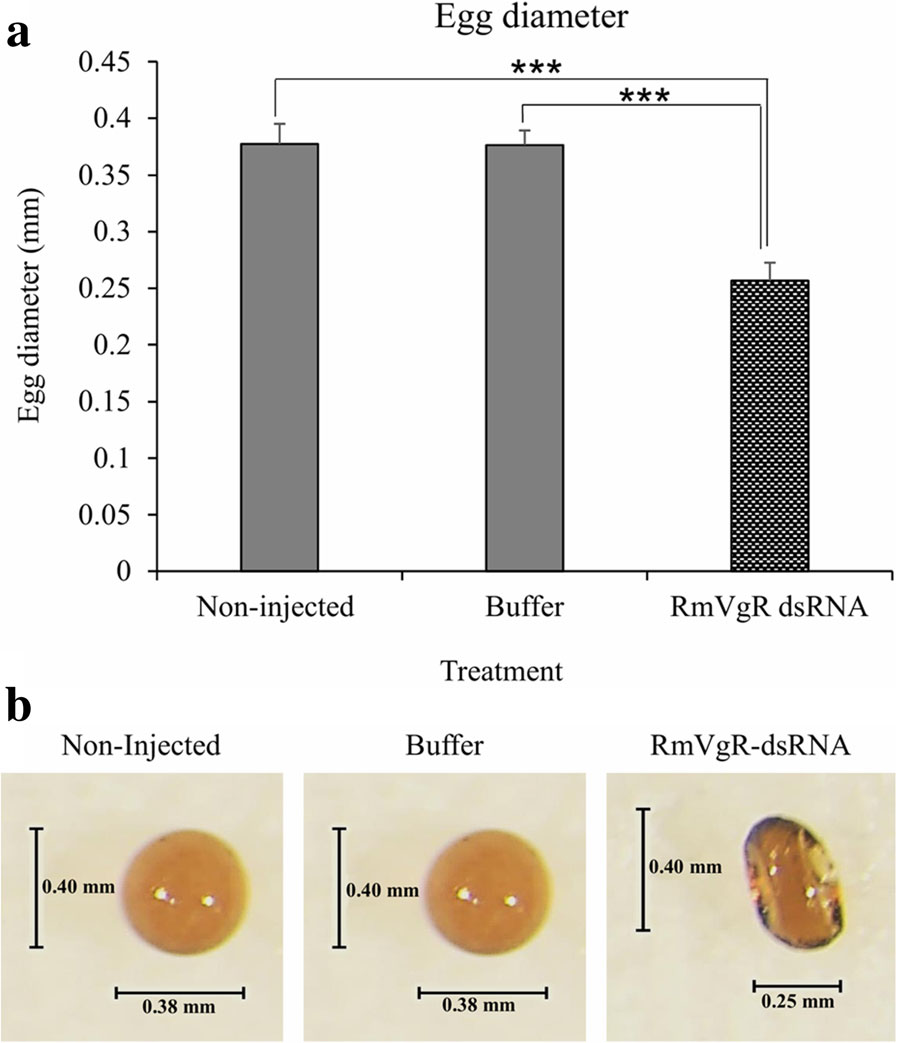
RmVgR dsRNA affected production of normal tick eggs. **a** Tick eggs diameter changed due to RmVgR silencing. **b** Individual egg measurement from the treatment groups. Asterisks indicate statistically significant differences: *t*_(60)_ = 98.5, *P* < 0.0001 (RmVgR-dsRNA-injected group *vs* buffer-injected group); and *t*_(60)_ = 29.3, *P* < 0.0001 (RmVgR-dsRNA-injected group *vs* non-injected group)

**Fig. 5:**
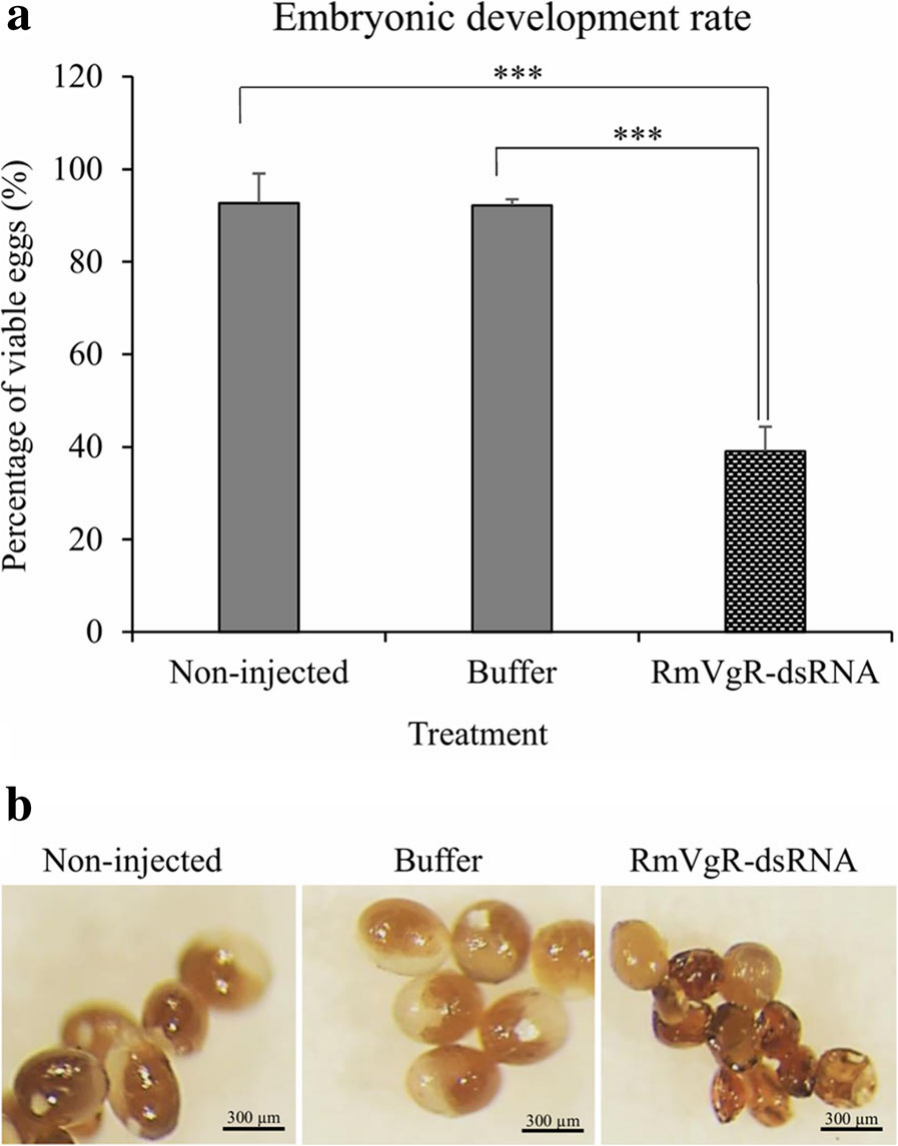
RmVgR dsRNA reduced tick egg embryonic development rate. **a** Tick egg embryonic development rate changed due to RmVgR silencing. **b** Embryonated eggs from the different treatment groups. Asterisks indicate statistically significant differences: *t*_(58)_ = 9.9, *P* < 0.0001 (RmVgR dsRNA-injected group *vs* buffer-injected group); and *t*_(58)_ = 10.4, *P* < 0.0001 (RmVgR dsRNA-injected group *vs* non-injected group)

More than 90% of female ticks in all three groups laid eggs. However, there were statistically significant differences in the average weight of the egg masses between tick groups (*P* < 0.0001) (Fig. 3) (Table 1). The average egg diameter in non-injected and buffer control groups were 0.379 mm and 0.376 mm, respectively (Table 1). In contrast, the average egg diameter in the RmVgR-dsRNA-injected group was 0.256 mm, a significant difference was found between groups (*P* < 0.0001) (Fig. 4a, b). We also observed a statistically significant difference in egg viability between RmVgR-dsRNA injected females as compared to non-injected and buffer control ticks. Control ticks had viabilities of 93% and 92%, respectively, whereas 39.1% of egg masses were viable from the dsRNA-injected group (*P* < 0.0001) (Fig. 5a, b).

There was no significant difference between groups in infection of adult female ticks with *B. bovis*. The overall female infection rate was around 70% in all groups demonstrating that RmVgR silencing did not affect *B. bovis* acquisition during tick feeding (Table 2). However, the rates of larval infection with *B. bovis* were 12% (7/58) and 17% (10/58) for non-injected and buffer control groups, respectively. In contrast, there were no larvae infected with *B. bovis* from the RmVgR-dsRNA group (*P* < 0.005), confirming that transovarial transmission of *B. bovis* to the offspring was diminished (Table 2).

**Table 2:** *Rhipicephalus microplus* VgR silencing effects in *Babesia bovis* infection of female ticks and parasite transovarial transmission to next generation of ticks

Group	Female tick infection (%)	Larvae infection rate (%)
Non-injected	67 (39/58)	12 (7/58)
Buffer	71 (41/58)	17 (10/58)
RmVgR-dsRNA	70 (40/58)	0 (0/58)

## Discussion

In a previous report, the phenotypic changes in the ovary from ticks of the RmVgR silenced group indicated that deposition of yolk is mediated by VgR [14]. Similar results, using a different region of VgR gene for silencing, have been reported for *A. hebraeum* and *R. microplus* where injection with VgR-dsRNA disturbed yolk deposition and reduced tick reproduction [15]. That data showed silencing of RmVgR impaired tick fertility, supporting a crucial role for VgR in the uptake of yolk protein by the ovary and in aiding egg formation during tick reproduction. Our data from the RmVgR-dsRNA-injected group corroborates the previous report and confirms that silencing RmVgR reduces tick reproductive fitness.

In the present study, we tested the hypothesis that silencing of vitellogenin receptor gene expression in the ovary during tick feeding on *B. bovis* infected cattle would affect parasite transmission to the next generation of ticks. Under normal conditions *B. bovis* is transmitted through infection of ovary epithelial cells by kinetes, resulting in transovarial transmission to tick eggs. Within the larvae, *B. bovis* develops in the salivary glands and transforms into sporozoites, an infectious stage for the mammalian host.

In this study, downregulation of VgR reduced transovarial transmission of *B. bovis*. As expected, due to the fact that VgR is expressed mainly by ovarian cells, there was no difference in female infection rate of *B. bovis* as determined by the presence of kinetes in tick hemolymph. VgR knockdown had no impact on adult female tick acquisition of *B. bovis* from the infected animal, as demonstrated by accumulation of kinetes in the tick hemolymph. These results, together with the success of female repletion, suggest that RmVgR-dsRNA had no effect on tick midgut epithelial cells. However, although a high female tick infection rate was found in the RmVgR-dsRNA group, the parasite was not transovarially transmitted to the next tick generation. These results are similar to the previous report using *H. longicornis* VgR (HlVgR) [14], in which silencing of HlVgR transcripts by RNAi caused abnormal oocyte development and led to atypical eggs and blocked transovarian transmission of *B. gibsoni* [14].

We postulate that VgR may be a potential mechanism for *B. bovis* kinete attachment and invasion of tick ovary epithelial cells resulting in infection of the next generation of ticks by transovarial transmission. One possible mechanism is that *B. bovis* kinete attach directly to the VgR on the ovary epithelial cells; an alternative mechanism is that kinete become coated with Vg which then facilitates indirect attachment to the Vg receptor on ovary epithelial cells. Based on these, and previously reported results, we are generating RmVgR specific synthetic peptides in order to formulate an experimental vaccine which will be tested in bovines in order to test whether RmVgR antibodies in vaccinated animals may affect tick fitness and parasite transmission.

## Conclusions

We have demonstrated that RmVgR silencing critically reduced oocyte development and egg maturation, resulting in decreased tick reproduction fitness. Moreover, it appears that RmVgR silencing directly reduced transovarial transmission of *B. bovis*. This conclusion was supported by the absence of *B. bovis* infected larvae from the RmVgR-dsRNA group. Therefore, RmVgR may be a good potential target for the development of novel strategies to control *R. microplus* and babesiosis by reducing tick reproduction and blocking pathogen transmission.

## Methods

### Synthesis of double stranded RNA

Synthesis of dsRNA was performed as previously described [17]. Specific primers were designed based on the RmVgR gene sequence (GenBank: KR401221) described in the *R. microplus* Gene Index Project [18] (Table 3). The sequence on GenBank (KR401221) was analyzed *in silico* for the identification of a sequence that is optimal for performing small interfering RNA (siRNA) using the algorithm siRNA Target Finder available from Ambion (Austin, TX, USA). A fragment of 361 bp from RmVgR gene containing the highest number of putative siRNA was identified as optimal and amplified by PCR and cloned in to PCR II-Topo (Invitrogen, Foster City, CA, USA) and used as a template for dsRNA synthesis. Reverse transcription PCR (RT-PCR) was used to amplify the RmVgR fragment from ovary mRNA. In brief, tick ovaries were removed with fine-tipped forceps and homogenized in TRIzol® reagent (Invitrogen). Total RNA was extracted following the manufacturer’s protocol and treated with DNase I (Invitrogen). Total RNA yield was quantified by Nanodrop (Thermo Fisher Scientific, Waltham, MA, USA). One hundred-fifty ng samples of total RNA were utilized to synthesize cDNA using the Superscript III kit (Thermo Fisher Scientific, Waltham, MA, USA) according to the manufacturer’s instructions, and the cDNA stored at -20 °C. The amplified fragment was at the 5' end of the RmVgR gene and approximately 361 bp in length. PCR products were cloned into pCR™II-TOPO® (Invitrogen), sequenced and used for *in vitro* transcription. The MEGAscript® Transcription Kit (Ambion) was used for dsRNA synthesis following the manufacturer’s protocol. The RmVgR dsRNA molecules were confirmed by electrophoresis on agarose gel, quantified by spectrophotometry and kept at -20 ^o^C until used for tick injection.

**Table 3:** Primer purposes and sequences, and PCR product size for *Rhipicephalus microplus VgR* silencing experiment

Primer name	Forward primer (5'-3')	Reverse primer (5'-3')	Product size (bp)
RmVgR-dsRNA	cgatgaagtcggctgtgatta	accaggcaatgcattcatgg	361
Rm-VgR	attgcgcagatttctcggac	ccgggagttgcacattcatc	181
Rm tub	cgtgccgtatttgttgatc	agattagctgctccgggtg	91
*B. bovis* rap1-Ex	cacgaggaaggaactaccgatgttga	ccaaggagcttcaacgtacgaggtca	354
*B. bovis* rap1-In	tcaacaaggtactctatatggctacc	ctaccgagcagaaccttcttcaccat	291

### Cattle, ticks and protozoan

A splenectomized Holstein calf (C90979) 3–4 months of age, that tested negative for *B. bovis* by PCR [17] and cELISA [19], was used in this study. The animal was maintained according to protocols approved by the University of Idaho Institutional Animal Care and Use Committee. Ticks from our laboratory colony, *R. microplus* La Minita strain [20] were used. To obtain unfed adult ticks for the experiment, approximately 40,000 larvae from 2 g of eggs were placed under a cloth patch to feed. On day 13, engorged nymphs were manually removed and held in an incubator at 25 ^o^C with 96% relative humidity to molt to adults.

### RmVgR-dsRNA injection

After 3 days of incubation, freshly molted adult ticks were sorted by sex. Three groups of 100 female ticks were used in this study, 1) non-injected control group, 2) buffer 1× phosphate-buffered saline (PBS)-injected group and 3) RmVgR-dsRNA-injected group. In brief, individual female ticks were injected through the coxal membrane at the base of leg 4 on the right ventral side, as previously described [17, 21] with 1 μl containing either ~1 × 10^11^ dsRNA molecules or buffer control. The injections were accomplished using a 10 μl syringe with a 33 gauge needle (Hamilton, Bonaduz, Switzerland) and a microprocessor controlled UMP3 injection pump apparatus (World Precision Instruments, Berlin, Germany). Each group was placed, with an equal number of males, under separate stockinet sleeves glued to the back of the calf to feed. One day after applying ticks, the calf was inoculated with *B. bovis* S_74_T_3_Bo strain [22] stabilate containing approximately 1.4 × 10^7^ *B. bovis*-infected erythrocytes so that the feeding of adult female ticks to repletion would be synchronized with rising *B. bovis* parasitemia (acquisition feeding). The infected calf was monitored daily for the presence of *B. bovis* in peripheral blood and clinical signs of babesiosis. Parasitemia of *B. bovis* in peripheral blood was examined by stained blood smears and PCR as previously described [17].

### Transcriptional analysis

Silencing of *RmVgR* after dsRNA injection was investigated using RT-PCR. Twenty-four partially and fully engorged adult female ticks were manually collected per group from the *B. bovis* infected calf, rinsed with 70% ethanol and dissected with sterile scalpel blades. Tick ovaries were harvested and total RNA extracted. cDNA was synthesized and PCR performed using RmVgR and Rm α-tubulin primers (Table 3) as previously described [23]. PCR amplicons were separated by electrophoresis on 2% agarose gels and visualized under UV trans-illumination. Amplicons were TA cloned into PCR 2.1-TOPO® (Thermo Fisher Scientific) and submitted for sequencing (Eurofins MWG Operon, Louisville, KY).

### Detection of *Babesia bovis* kinetes

Fully engorged female ticks (*n* = 58 per group) from non-injected, buffer-injected, and RmVgR-dsRNA-injected groups were examined to determine tick infection rates as previously described [22], In brief, hemolymph from individual ticks was sampled on day 8 after dropping. A distal leg segment was removed, and a drop of exuded hemolymph was placed onto a glass slide and stained with Giemsa as previously described [23] for the presence of *B. bovis* kinetes. A minimum of 50 high-power fields per sample were observed by light microscopy.

### Evaluation of tick fitness

Fully engorged female ticks were collected, weighed and put in individual wells in 24-well plates at 26 ^o^C. After 14 days of incubation, female ticks began oviposition. After completion of oviposition egg masses from individual female ticks were weighed and placed in individual vials. The embryonic development rate was determined by the percentage of eggs with an embryo per female. Effects derived from the knockdown of the VgR were estimated by measuring egg diameter, size and viability using a Leica MZ12.5 microscope. Pictures were taken by SPOT Insight 11.2 Color Mosaic (Diagnostic Instruments, Inc.) and analyzed by SPOT 5.2 Software (Diagnostic Instruments, Inc.).

### Detection of *Babesia bovis* transovarial transmission

Whole-genomic DNA was isolated from individual unfed *R. microplus* larva from the three groups. Larvae were collected, washed in 75% ethanol, and stored at -20 ^o^C. Frozen individual larva were triturated in 20 μl phosphate-buffered saline (pH=7.2) and the homogenate transferred into a 1.5 ml microcentrifuge tube to which 300 μl of cell lysis buffer (Puregene, Valencia, CA, USA) and 15 μl of Proteinase K (2 mg/ml stock) was added and vortexed. The homogenate was incubated at 56 °C overnight, then 200 μl cold Protein Precipitation Solution (Puregene) was added to the lysate, vortexed and incubated on ice for 5 min. The lysate was centrifuged at 13,200× *rpm* for 3 min. The supernatant was poured to a clean screw-cap 1.5 ml tube with 500 μl isopropanol and mixed by inverting 50 times. After incubation at room temperature for 10 min, the genomic DNA was precipitated by centrifugation at 13,200× *rpm* for 5 min. Thereafter, 2 washes were performed with 300 μl of 70% ethanol at 13,200× *rpm* for 5 min. The pellets were air dried for 10 min and suspended with 30 μl of TE buffer and stored at -20 ^o^C.

Nested primer sets were used to amplify the *B. bovis rap1* gene (Table 3) using the previously described method [24]. PCR products were separated by electrophoresis on 2% agarose gels and visualized with UV trans-illumination. All the PCR amplicons were cloned into PCR 2.1-TOPO® (Thermo Fisher Scientific) and submitted for sequencing (Eurofins MWG Operon, Louisville, KY).

### Statistical analysis

Weight of egg masses, egg diameter, embryonic development rate and parasite infection rate were compared by Student’s t-test (GraphPad Instat®, version 3.06, GraphPad Software, Inc., San Diego, CA, USA).

## Abbreviations

dsRNA: Double stranded RNA
PBS: Phosphate-buffered saline
RmVgR: *Rhipicephalus microplus* vitellogenin receptor
RNAi: RNA interference
RT-PCR: Reverse transcription PCR
Vg: Vitellogenin
VgR: Vitellogenin receptor

## References

[1] Dantas-Torres F, Chomel BB, Otranto D (2012). Ticks and tick-borne diseases: a One Health perspective. Trends Parasitol..

[2] Roberts JA (1968). Resistance of cattle to the tick *Boophilus microplus* (Canestrini). II. Stages of the life cycle of the parasite against which resistance is manifest. J Parasitol..

[3] Grisi L, Leite RC, JRdS M, ATMd B, Andreotti R, PHD C (2014). Reassessment of the potential economic impact of cattle parasites in Brazil. Rev Bras Parasitol Vet..

[4] Bergamo Estrela A, Seixas A, de Oliveira Nunes Teixeira V, AFM P, Termignoni C (2010). Vitellin- and hemoglobin-digesting enzymes in *Rhipicephalus* (*Boophilus*) *microplus* larvae and females. Comp Biochem Physiol B Biochem Mol Biol.

[5] Rosell R, Coons LB (1992). The role of the fat body, midgut and ovary in vitellogenin production and vitellogenesis in the female tick *Dermacentor variabilis*. Int J Parasitol..

[6] Thompson DM, Khalil SMS, Jeffers LA, Sonenshine DE, Mitchell RD, Osgood CJ, Michael Roe R (2007). Sequence and the developmental and tissue-specific regulation of the first complete vitellogenin messenger RNA from ticks responsible for heme sequestration. Insect Biochem Mol Biol..

[7] Boldbaatar D, Umemiya-Shirafuji R, Liao M, Tanaka T, Xuan X, Fujisaki K (2010). Multiple vitellogenins from the *Haemaphysalis longicornis* tick are crucial for ovarian development. J Insect Physiol..

[8] Khalil SMS, Donohue KV, Thompson DM, Jeffers LA, Ananthapadmanaban U, Sonenshine DE (2011). Full-length sequence, regulation and developmental studies of a second vitellogenin gene from the American dog tick, *Dermacentor variabilis*. J Insect Physiol.

[9] Xavier MA, Tirloni L, Pinto AFM, Diedrich JK, Yates JR, Mulenga A (2018). A proteomic insight into vitellogenesis during tick ovary maturation. Sci Rep..

[10] Barrero RA, Guerrero FD, Black M, McCooke J, Chapman B, Schilkey F (2017). Gene-enriched draft genome of the cattle tick *Rhipicephalus microplus*: assembly by the hybrid Pacific Biosciences/Illumina approach enabled analysis of the highly repetitive genome. Int J Parasitol..

[11] Giorgi F, Bradley JT, Nordin JH (1999). Differential vitellin polypeptide processing in insect embryos. Micron..

[12] Raikhel AS, Dhadialla TS (1992). Accumulation of yolk proteins in insect oocytes. Annu Rev Entomol..

[13] Mitchell Iii RD, Ross E, Osgood C, Sonenshine DE, Donohue KV, Khalil SM (2007). Molecular characterization, tissue-specific expression and RNAi knockdown of the first vitellogenin receptor from a tick. Insect Biochem. Mol Biol..

[14] Boldbaatar D, Battsetseg B, Matsuo T, Hatta T, Umemiya-Shirafuji R, Xuan X, Fujisaki K (2008). Tick vitellogenin receptor reveals critical role in oocyte development and transovarial transmission of *Babesia* parasite. Int J Biochem Cell Biol..

[15] Smith AD, Reuben Kaufman W (2013). Molecular characterization of the vitellogenin receptor from the tick, *Amblyomma hebraeum* (Acari: Ixodidae). Insect Biochem. Mol Biol..

[16] Seixas A, Alzugaray MF, Tirloni L, Parizi LF, Pinto AFM, NWo G (2018). Expression profile of *Rhipicephalus microplus* vitellogenin receptor during oogenesis. Ticks Tick Borne Dis..

[17] Bastos RG, Ueti MW, Guerrero FD, Knowles DP, Scoles GA (2009). Silencing of a putative immunophilin gene in the cattle tick *Rhipicephalus* (*Boophilus*) *microplus* increases the infection rate of *Babesia bovis* in larval progeny. Parasit Vectors..

[18] Guerrero FD, Miller RJ, Rousseau ME, Sunkara S, Quackenbush J, Lee Y, Nene V (2005). BmiGI: A database of cDNAs expressed in *Boophilus microplus*, the tropical/southern cattle tick. Insect Biochem Mol Biol..

[19] Goff WL, Molloy JB, Johnson WC, Suarez CE, Pino I, Rhalem A (2006). Validation of a competitive enzyme-linked immunosorbent assay for detection of antibodies against *Babesia bovis*. Clin Vaccine Immunol..

[20] Stiller D, Goff WL, Johnson LW, Knowles DP (2002). *Dermacentor variabilis* and *Boophilus microplus* (Acari: Ixodidae): experimental vectors of *Babesia equi* to equids. J Med Entomol.

[21] Hussein HE, Scoles GA, Ueti MW, Suarez CE, Adham FK, Guerrero FD, Bastos RG (2015). Targeted silencing of the Aquaporin 2 gene of *Rhipicephalus* (*Boophilus*) *microplus* reduces tick fitness. Parasit Vectors..

[22] Rodriguez SD, Buening GM, Green TJ, Carson CA (1983). Cloning of *Babesia bovis* by *in vitro* cultivation. Infect Immun.

[23] Ueti MW, Palmer GH, Kappmeyer LS, Scoles GA, Knowles DP (2003). Expression of Equi Merozoite antigen 2 during development of *Babesia equi* in the midgut and salivary gland of the vector tick *Boophilus microplus*. J Clin Microbiol..

[24] Figueroa JV, Chieves LP, Johnson GS, Buening GM (1993). Multiplex polymerase chain reaction based assay for the detection of *Babesia bigemina, Babesia bovis* and *Anaplasma marginale* DNA in bovine blood. Vet Parasitol..

